# Surgical Treatment for Malunion of the Lateral Humeral Epicondyle with Posterior Subluxation of the Radial Head: A Case Report and Literature Review

**DOI:** 10.1155/2018/1901235

**Published:** 2018-07-24

**Authors:** Koichi Yano, Yasunori Kaneshiro, Hideki Sakanaka

**Affiliations:** Department of Orthopaedic Surgery, Seikeikai Hospital, 1-1-1 Minamiyasuicho, Sakai-ku, Sakai, Osaka 590-0064, Japan

## Abstract

A 24-year-old right-handed man suffered right olecranon and lateral epicondylar fracture from high energy trauma. Fixation of olecranon was performed by a previous doctor. Three months after operation, he presented with limited range of motion (ROM) of the right elbow caused by malunion of the lateral epicondylar fracture and subluxation of the radiohumeral joint. Preoperative ROM of the right elbow was flexion 110° and extension −75°. Forearm rotation was pronation 85° and supination 65°. Fragment excision of the lateral epicondyle, which was 27 mm in length, and lateral collateral ligament repair using anchors were performed. Fourteen months postoperatively, contracture release of the elbow was performed. Twenty-four months postoperatively, radiograph of the elbow showed normal congruence without osteoarthritic changes and the ROM of the right elbow was flexion 120° and extension −35°. Forearm rotation was pronation 90° and supination 70°. In the surgical setting, in case of the size of the lateral epicondylar fragment is relatively large, the fragment should be fixed or lateral collateral ligament should be repaired when the instability of the elbow is found.

## 1. Introduction

The lateral collateral ligament (LCL) of the elbow forms complexes and is composed of the radial collateral ligament, the lateral ulnar collateral ligament (LUCL), annular ligament, and accessory LCL [1–3]. The wrist and finger extensors and the LCL complex insert onto the lateral epicondyle of the humerus [2]. When the LUCL and other soft tissues have disrupted, posterolateral rotatory instability (PLRI) of the elbow occurs [1–3]. Fracture with PLRI (i.e., lateral humeral epicondyle fracture) is rare; moreover, a chronic condition is a very rare entity [4, 5]. We treated a patient who presented with a limited range of motion of the elbow. He had a chronic osseous avulsion fracture of the lateral epicondyle that had displaced posterolaterally and included the entire insertion of the LCL and extensor muscles to the humerus.

The purpose of this case study was to report the surgical outcomes of a patient with malunion of the avulsed lateral humeral epicondyle and subluxation of the humeroradial joint.

## 2. Case Presentation

**Figure 1:**
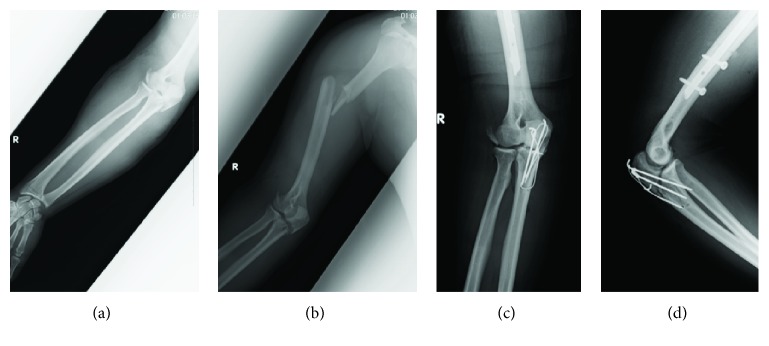
Radiographs of the injury and first surgery at the previous hospital. (a) Anteroposterior (AP) view of the forearm and elbow. (b) AP view of the humerus and elbow. Diaphyseal fracture of humerus, olecranon fracture, and lateral epicondylar fracture are found. (c) AP view and (d) lateral view of the elbow after surgery. The olecranon is fixed by tension band wiring. The lateral epicondylar fracture is displaced with normal joint congruence.

Informed consent for publication was obtained from the patient. A 24-year-old right-handed man fell from the fifth floor balcony and was transported to an emergency hospital. Radiological examinations revealed that he had suffered right traumatic pneumothorax, right humeral shaft fracture, right olecranon fracture, right scapular fracture, and right radial nerve palsy (Figures 1(a) and 1(b)). All fractures were closed. After respiratory system stabilization, the right olecranon and humeral shaft were operated on 11 days after the injury (Figures 1(c) and 1(d)). Three months after the injury, he presented with limited range of motion (ROM) of the elbow and persistent radial nerve palsy.

Physical examination showed grip strengths (measured with a digital dynamometer) of 7.3 kg and 39.0 kg in the right and left hand, respectively. The respective ROM for the right and left extremities (measured with a standard goniometer) was as follows: elbow flexion, 110° and 140°; elbow extension, −75° and 0°; forearm pronation, 85° and 85°; and forearm supination, 65° and 90°. Muscle strength was M4 for the right triceps and M1 for the wrist and finger extensors. Sensory disturbance (3/10 on the ten test) was observed in the area of the radial nerve. The PLRI test result was negative.

**Figure 2:**
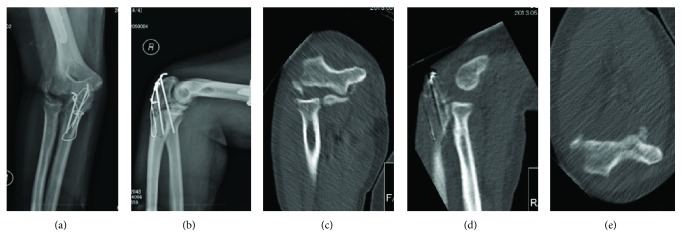
Radiographs and computed tomography (CT) image 3 months after surgery. (a) AP view and (b) lateral view of the elbow. The avulsed fracture of the lateral epicondyle is displaced, the radiohumeral joint is widened in the AP view, and the posterior subluxation of the radial head is widened in the lateral view. The joint congruency of the ulnohumeral joint is maintained. (c) CT image in the coronal plane, (d) sagittal view, and (e) axial view. The displaced lateral epicondylar fracture is malunited.

Plain radiography and computed tomography at three months after the injury showed that the avulsion fracture of the lateral epicondyle became displaced and malunited, the radiohumeral joint had widened on the anteroposterior view, and the posterior subluxation of the radial head had widened on the lateral view (Figures 2(a)–2(e)). We diagnosed subluxation of the radial head caused by malunion of the lateral humeral epicondyle and incomplete palsy of the radial nerve. Conservative treatment was selected for recovery of the radial nerve palsy.

**Figure 3:**
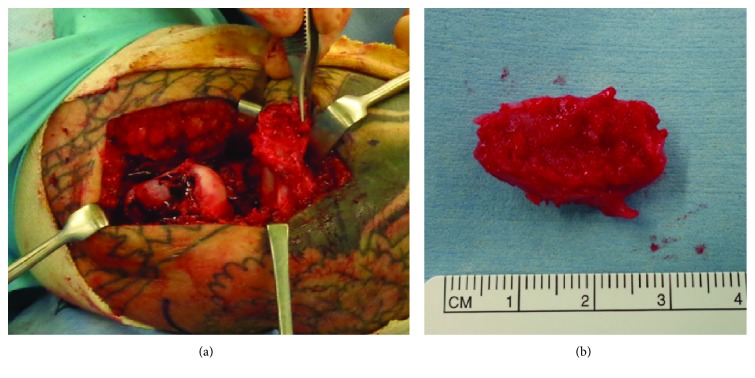
Intraoperative images. (a) After osteotomy of the displaced lateral epicondylar fracture, the fragment contains lateral collateral ligament and extensor muscles. (b) The resected fragment. The size of the fragment is 27 mm in length.

Surgery was performed under general anesthesia. A lateral approach was used. The avulsed lateral epicondyle was malunited and detached from the original site. The malunited lateral epicondylar fragment was osteotomized by a chisel. The fragment contained the origin of the LCL complex and extensor muscles (Figure 3(a)). The cartilage of the capitellum and radial head was not injured. Because anatomical reduction of the fragment was impossible, the bony fragment was resected under the periosteum. The fragment size was 27 mm in length (Figure 3(b)). After the LCL complex was pulled tight, the quality of the LCL was judged as good without cheese cut. The isometric point of the LCL was confirmed during elbow flexion-extension, and the LCL successfully reached this point. The LCL was repaired using two anchors. Extensor muscles were repaired to the surrounding soft tissue. The elbow was immobilized with a splint with an elbow flexion of 90° and full pronation. Two days after surgery, the elbow was immobilized with a long arm cast in the same position. Three weeks after surgery, the elbow was fixed with a hinged brace at the forearm position in pronation. The extension of the elbow was limited to −30°. Six weeks after surgery, extension limitation reduced to 0° and forearm rotation exercises were started. Three months after surgery, the brace was removed. Six months after surgery, all range of activity was permitted.

Because the patient complained of limited elbow ROM (elbow flexion, 110°; extension, −50°) and irritation from the olecranon hardware, a second operation was performed 14 months after surgery. Under general anesthesia, the olecranon hardware was removed, the anterior and posterior capsules were released, and the posterior oblique ligament of the medial collateral ligament of the elbow was resected via a medial approach. Flexion ROM of 135° and extension ROM of −30° were achieved intraoperatively.

**Figure 4:**
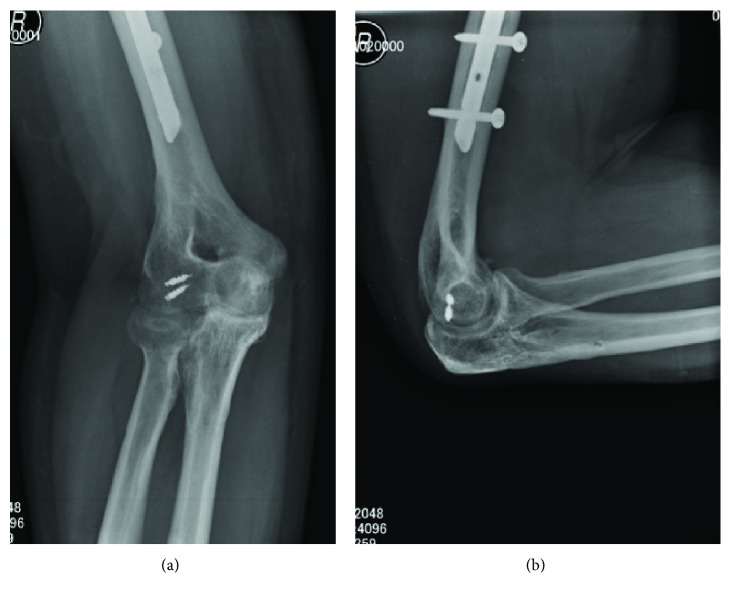
Radiographs 2 years after surgery. (a) AP view and (b) lateral view. Humeroradial joint congruency is good without osteoarthritic change.

At the final follow-up 2 years after surgery, the motor and sensory palsy of the radial nerve recovered to levels of the healthy side and grip strength for the right and left hands were 35.9 kg and 37.3 kg, respectively. The respective ROM for the right extremity was as follows: elbow flexion, 120°; elbow extension, −35°; forearm pronation, 90°; and forearm supination, 70°. The radiographs showed normal congruence of the elbow joint without osteoarthritic change (Figures 4(a) and 4(b)).

## 3. Discussion

We presented a case of olecranon and lateral humeral epicondyle fracture caused by high energy trauma. Initial treatment involved fixation of the olecranon, resulting in limited ROM of the elbow caused by malunion of the lateral humeral epicondyle. This pathology was caused by the displaced and malunited lateral epicondyle, including the entire insertion of the LCL and the extensors, which contribute to stability of the lateral side of the elbow [2].

In the previous literature, the concomitant conditions of lateral humeral epicondyle fracture and LCL function insufficiency in adults have been reported in two case reports. In the acute setting, a 16-year-old man had suffered lateral epicondyle fracture and PLRI on initial presentation [4]. This case was treated by osteosynthesis of the lateral epicondyle and repair of the LCL, and bone union and stability of the elbow joint were achieved. In the chronic setting, a 36-year-old woman presented 8 months after injury with nonunion of the lateral humeral epicondyle [5]. Instability of the elbow was confirmed under general anesthesia. After resection of the epicondylar fragment, the LCL was repaired. At the 2-year follow-up, stability of the elbow joint had been achieved without arthritic changes. Both reports achieved stability of the elbow by osteosynthesis or ligament repair. In our case, the lateral epicondylar fragment included the LCL and extensors and should have been fixed during the initial treatment after checking instability.

We used cast immobilization instead of early mobilization, possibly accounting for the limited range of elbow motion postoperatively.

To the best of our knowledge, there has been no report of a case series with chronic lateral epicondylar nonunion or malunion. On the other hand, there are some reports on the surgical outcomes of instability due to medial epicondylar nonunion. Gilchrist et al. reported 5 cases of fragment excision of the medial epicondylar fragment and medial collateral ligament repair using suture anchors [6]. The average age of patients was 35 years, and the average duration from injury to surgery was 10 years. The ROM of the flexion-extension arc of the elbow at average 22 months after surgery was 117° without instability. Kaneshiro et al. reported the clinical results of excision of the medial epicondyle and ligament reconstruction using tendon grafts in 5 cases [7]. The average age of patients was 36 years, and the average duration from injury to surgery was 25 years. The ROM of the flexion-extension arc of the elbow at average 10 months after surgery was 138° without pain and instability. Ligament reconstruction with tendon grafts seems to yield better results because of the replacement of the shortened ligament. Because the final ROM of the elbow was still limited in our case in spite of repairing the ligament by judging its quality, a better ROM may have been obtained with ligament reconstruction.
